# Area-level global and local clustering of human *Salmonella* Enteritidis infection rates in the city of Toronto, Canada, 2007–2009

**DOI:** 10.1186/s12879-015-1106-6

**Published:** 2015-08-21

**Authors:** Csaba Varga, David L. Pearl, Scott A. McEwen, Jan M. Sargeant, Frank Pollari, Michele T. Guerin

**Affiliations:** Department of Population Medicine, Ontario Veterinary College, University of Guelph, Guelph, ON N1G 2W1 Canada; Ontario Ministry of Agriculture, Food and Rural Affairs, Guelph, ON N1G 4Y2 Canada; Centre for Public Health and Zoonoses, Ontario Veterinary College, University of Guelph, Guelph, ON N1G 2W1 Canada; Centre for Foodborne, Environmental and Zoonotic Infectious Diseases, Public Health Agency of Canada, Guelph, ON N1H 8J1 Canada

## Abstract

**Background:**

*Salmonella enterica* serotype Enteritidis (*S.* Enteritidis) remains a major foodborne pathogen in North America yet studies examining the spatial epidemiology of salmonellosis in urban environments are lacking. Our ecological study combined a number of spatial statistical methods with a geographic information system to assess area-level heterogeneity of *S.* Enteritidis infection rates in the city of Toronto.

**Methods:**

Data on *S.* Enteritidis infections between January 1, 2007 and December 31, 2009 were obtained from Ontario’s surveillance system, and were grouped and analyzed at the forward sortation area (**FSA**)-level (an area signified by the first three characters of the postal code). Incidence rates were directly standardized using the FSA-level age- and sex-based standard population. A spatial empirical Bayes method was used to smooth the standardized incidence rates (**SIRs**). Global clustering of FSAs with high or low non-smoothed SIRs was evaluated using the Getis-Ord G method. Local clustering of FSAs with high, low, or dissimilar non-smoothed SIRs was assessed using the Getis-Ord Gi* and the Local Moran’s I methods.

**Results:**

Spatial heterogeneity of *S.* Enteritidis infection rates was detected across the city of Toronto. The non-smoothed FSA-level SIRs ranged from 0 to 16.9 infections per 100,000 person-years (mean = 6.6), whereas the smoothed SIRs ranged from 2.9 to 11.1 (mean = 6.3). The global Getis-Ord G method showed significant (p ≤ 0.05) maximum spatial clustering of FSAs with high SIRs at 3.3 km. The local Getis-Ord Gi* method identified eight FSAs with significantly high SIRs and one FSA with a significantly low SIR. The Local Moran’s I method detected five FSAs with significantly high-high SIRs, one FSA with a significantly low-low SIR, and four significant outlier FSAs (one high-low, and three low-high).

**Conclusions:**

*Salmonella* Enteritidis infection rates clustered globally at a small distance band, suggesting clustering of high SIRs in small distinct areas. This finding was supported by the local cluster analyses, where distinct FSAs with high SIRs, mainly in downtown Toronto, were detected. These areas should be evaluated by future studies to identify risk factors of disease in order to implement targeted prevention and control programs. We demonstrated the usefulness of combining several spatial statistical techniques with a geographic information system to detect geographical areas of interest for further study, and to evaluate spatial processes that influenced *S.* Enteritidis infection rates. Our study methodology could be applied to other foodborne disease surveillance data.

**Electronic supplementary material:**

The online version of this article (doi:10.1186/s12879-015-1106-6) contains supplementary material, which is available to authorized users.

## Background

Salmonellosis continuously poses a significant health burden to human populations globally, affecting annually an estimated 93.8 million persons worldwide [1]. In Canada, an estimated 109,384 non-typhoidal *Salmonella* infections are acquired domestically, of which 80 % are considered to be foodborne [2]. Within the last decade, an increase in the number of *Salmonella enterica* serotype Enteritidis (*S.* Enteritidis) infections has been reported in Canada [3], the United States of America [4], and the European Union [5], such that *S.* Enteritidis has become the top serotype among the non-typhoidal salmonellae. *Salmonella* Enteritidis infections in humans have typically been associated with consumption of contaminated chicken products [6, 7] and eggs [8, 9]. However, salmonellosis has recently been linked to other factors, including international travel [10, 11], demographic [12, 13] and socioeconomic [14, 15] characteristics, and animal contact [7, 16].

Country- or region-level studies have used various spatial epidemiological methods to identify clustering of health conditions, including notifiable gastrointestinal illness [17], giardiasis [18], campylobacteriosis [19, 20], influenza B [21], *Escherichia coli* O157 [22, 23], dengue fever [24, 25], traumatic brain injury [26], stroke [27], and myocardial infarction [27]. Moreover, city-level studies have evaluated spatial differences in neighbourhood-level infection rates of rotavirus in Berlin, Germany [28], pandemic influenza A in Hong Kong [29], tuberculosis in Linyi City, China [30], and typhoid fever [31–33] and dengue [34] in the Dhaka metropolitan area of Bangladesh.

**Fig. 1:**
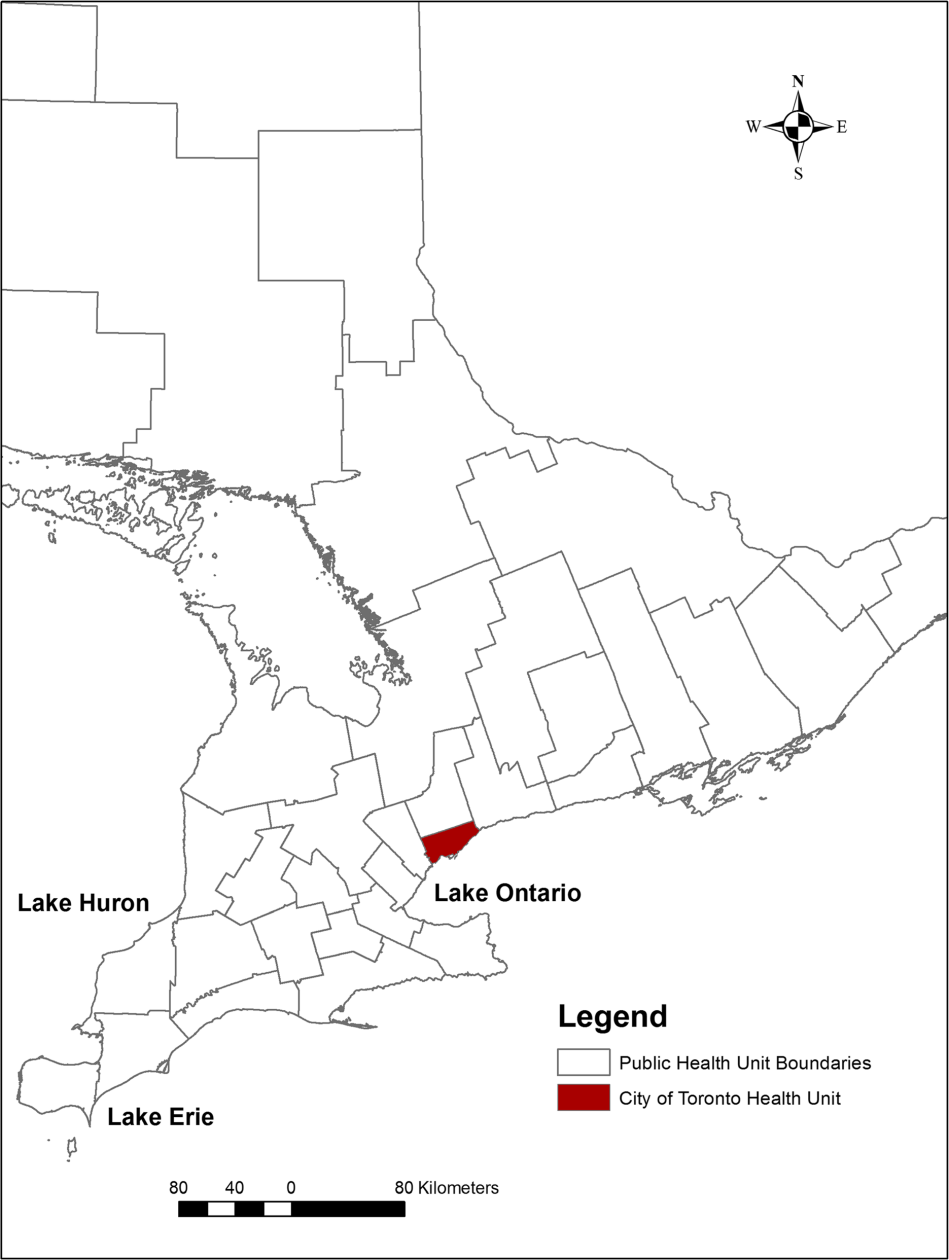
Map of Ontario, Canada highlighting the location of the study area

Our study area involved the city of Toronto—the capital of Ontario, Canada located on the shore of Lake Ontario in the southern part of the province (Fig. 1). In 2009, an estimated 2.7 million people lived in the city, accounting for 21 % of Ontario’s total population [35]. Toronto’s forward sortation areas (**FSAs**; areas signified by the first three characters of the postal code; see Study design and data sources section) have diverse age- and sex-based populations that can affect area-level infection rates, due to sex differences of salmonellosis rates [36, 37], and younger and older residents’ higher salmonellosis rates [13, 16, 38]. Standardization of area-level infection rates based on the age and sex distribution of the population has been recommended to overcome this problem [39]. Moreover, infection rates in small population areas can become unstable and unreliable. The spatial empirical Bayes (**SEB**) smoothing method has been proposed to reduce the random variation of infection rates linked with these areas [39, 40].

**Fig. 2:**
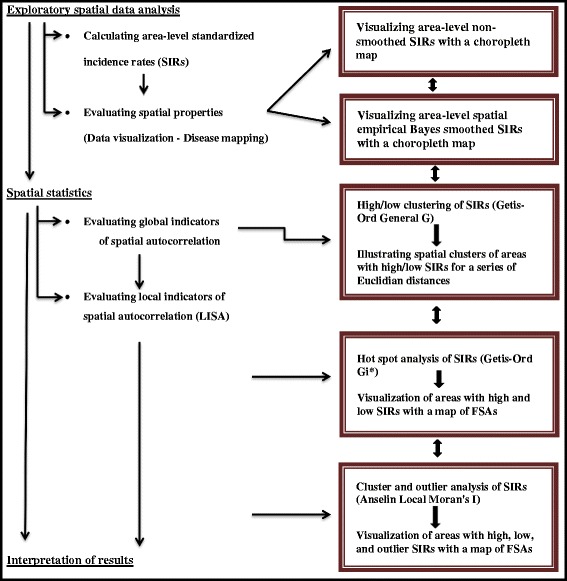
Flow chart outlining the analytical steps used to evaluate area-level *Salmonella* Enteritidis infection rates

Despite the abundance of research studies that have assessed large scale (country- or region-level) spatial processes that influence foodborne infections, few studies have assessed small scale (city- or FSA-level) spatial clustering of salmonellosis rates. Small area studies in urban environments are useful as a first step for identifying areas with high infection rates, where future studies can be conducted to identify novel individual-level risk factors, which can assist in the design of local prevention and control programs [21, 28]. Our retrospective, population-based, ecological study used a systematic approach that combined spatial exploratory and statistical methods with a geographic information system (**GIS**) (Fig. 2), to evaluate the spatial heterogeneity of *S.* Enteritidis infection rates across the city of Toronto. Moreover, two local spatial cluster detection methods were compared to identify their strengths and weaknesses in analyzing small-scale infectious disease data.

## Methods

### Study design and data sources

Forward sortation areas are well-delimited areas signified by the first three characters of the postal code; they are established by the Canada Post Corporation based on the mail distribution zones of postal facilities. Forward sortation area-level population estimates and FSA cartographical boundary files were acquired from the 2006 Census of Canada [35, 41].

In Ontario, salmonellosis is a reportable disease under provincial legislation [42]. A diagnosis of salmonellosis is made after isolation of *Salmonella* spp. (excluding *Salmonella* Typhi or Paratyphi) from an appropriate clinical sample (the majority are stool samples) by public health, hospital, or private laboratory staff [43]. All isolates are sent to the Toronto Public Health Laboratories for confirmation and serotyping using the Kauffmann-White scheme [44]. Salmonellosis cases must be followed up by local public health unit staff, and investigation findings must be reported to the Ontario Ministry of Health and Long-Term Care (MOHLTC) through the integrated Public Health Information System (iPHIS). This surveillance system is a repository for all reportable disease data in Ontario; no major modifications in salmonellosis reporting requirements, or testing or case follow-up protocols were noted during the study period, which makes salmonellosis case ascertainments robust and reliable.

We obtained case information from all reported *S.* Enteritidis infections from the city of Toronto between January 1, 2007 and December 31, 2009 that were captured within iPHIS.

### Statistical analysis

Spatial heterogeneity of *S.* Enteritidis infection rates was assessed by following several analytical steps, which are outlined in Fig. 2, and described in detail below.

### Exploratory spatial analysis

In order to obtain stable infection rate estimates, we excluded FSAs with less than 500 residents. Annual standardized incidence rates (**SIRs**) were calculated for each FSA using direct standardization [45, 46] in STATA Intercooled 10.1 statistical software (Stata Corporation, College Station, TX, USA). The annual SIR was estimated by calculating the observed rate for each age-sex category within each FSA, and multiplying it by the age-sex population numbers, which were obtained from the 2006 Census of Canada [35]. Age categories were in five-year increments from 0 to > 85 years [47]. To account for unstable SIRs of areas with small populations [48], we smoothed the rates using the SEB method [49] with 2^nd^ order queen contiguity weights [50] in GeoDa version 095i software (Spatial Analysis Lab, University of Illinois Urbana-Champaign, IL, USA). The non-smoothed and smoothed annual SIRs were presented as the number of *S.* Enteritidis infections per 100,000 person-years per FSA, and were visualized using choropleth maps with ArcGIS 10.1 (ESRI Inc., Redlands, CA, USA) using Jenk’s categorization [51] to define the critical intervals for mapping. Jenk’s natural breaks classification was developed to identify the ideal arrangement of values (*e.g.* rates) into different classes, by reducing the variance within classes and maximizing the variance between classes [51].

### Spatial statistics

Each FSA was represented by a polygon, its centroid, and its distinct non-smoothed SIR. The *Spatial Statistics Tool* in ArcGIS 10.1 was used to identify global and local spatial clusters. Euclidean distance bands were used to measure distances from each FSA’s centroid to neighbouring FSAs’ centroids (see Global clustering (Getis-Ord General G) subsection). To avoid the omission of local factors by imposing sharp neighbourhood boundaries, the “zone of indifference” conceptualization parameter was chosen for our global and local cluster analyses. Using this parameter, the target FSA and all neighbouring FSAs within a specified distance band are given a maximum weight; once this critical distance is exceeded, neighbouring FSAs are assigned smaller and smaller weights as the distance from the target FSA increases [52, 53]. The null hypothesis for both global and local cluster analyses is that there is complete spatial randomness (*i.e.* FSAs with high or low non-smoothed SIRs are randomly distributed across the study area). The null hypothesis is rejected when FSAs with high or low SIRs are more spatially clustered than would be expected if the underlying spatial processes were truly random. When the null hypothesis is rejected, a Z-score and a p-value are given for the identified cluster [52, 53].

#### Global clustering (Getis-Ord General G)

Global spatial clustering of FSAs with high or low non-smoothed SIRs across Toronto was evaluated using the Getis-Ord General G statistic [53]. Distance bands that required each FSA to have at least one neighbour were manually selected; for our data, the minimum distance band was 3.3 km. Several Euclidean distances (3.3 to 5.9 km, with 100 m increments) were selected and included in the model to identify the distance bands with the highest and lowest statistically significant Z-scores. A large, positive Z-score (values ≥ 1.96) and a significant p-value (*p* ≤ 0.05) signified that FSAs with high SIRs were clustered in the study area, whereas a large, negative Z-score (values ≤ -1.96) and a significant p-value signified that FSAs with low SIRs were clustered in the study area [53].

### Local clustering

For the local cluster analyses, we used the distance band identified at the global clustering step that showed maximum spatial clustering of FSAs with high non-smoothed SIRs (see Global clustering (Getis-Ord General G) subsection).

#### Hot spot analysis (Getis-Ord Gi*)

Local spatial clusters of FSAs with high or low non-smoothed SIRs were examined using the Getis-Ord Gi* statistic [53, 54]. The statistic compares the local sum of SIRs (the sum of the SIR of the targeted FSA and its neighbouring FSAs) to the sum of SIRs of all FSAs within the study area. A statistically significant large, positive Z-score signifies a local high-rate cluster (*hot spot*). Hot spots are detected when FSAs with high rates are surrounded by FSAs with high rates; the observed local sum of SIRs is higher than the expected local sum and the difference is too large to be the result of chance alone. Similarly, a statistically significant large, negative Z-score signifies a local low-rate cluster (*cold spot*), where FSAs with low rates are surrounded by FSAs with low rates [51–54]. Statistically significant hot and cold spots were visualized using a map with FSA boundaries.

### Cluster and outlier analysis (Anselin Local Moran’s I)

We also used the Local Moran’s I statistic to identify local spatial clusters of FSA-level non-smoothed *S.* Enteritidis SIRs during the study period [55]. The statistic identifies hot spots (high-high), cold spots (low-low), and spatial outliers (high-low and low-high). A positive Local Moran’s I value indicates that the target FSA is surrounded by FSAs with similar rates (*high-high*: FSA with a high rate surrounded by FSAs with high rates; *low-low*: FSA with a low rate surrounded by FSAs with low rates). A negative Local Moran’s I value indicates that the target FSA is surrounded by FSAs with dissimilar rates (*high-low*: FSA with a high rate surrounded by FSAs with low rates; *low-high*: FSA with a low rate surrounded by FSAs with high rates) [55]. The designation of FSAs to these four classes depends on the results of a statistical test. This test performs random comparisons among the target FSA’s and its neighbours Moran’s I values to all FSAs’ Moran’s I values within the study area, and compares the observed Moran’s I value to the value corresponding to the random permutations (expected Moran’s I value) [55]. If the test is significant (p ≤ 0.05), the observed Moran’s I value is significantly larger (or smaller in the case of a negative relationship) than the expected Moran’s I value. If the test is not significant, the FSA remains in a neutral class (no spatial dependence) [55]. Statistically significant high-high, low-low, and outlier local clusters were visualized using a map with FSA boundaries. The two local cluster analytical methods were compared to evaluate their efficacy in identifying local infection clusters (*e.g.* sensitivity analysis).

### Ethics review

The University of Guelph Ethics Review Board was consulted since we used surveillance data for a reportable disease of humans; however, ethics approval was not required because our data did not contain any personal or health information that could be connected back to the original identifiers.

## Results

### Descriptive statistics

Based on the 2006 Census, there were a total of 102 FSAs in the city of Toronto; the FSA-level population size ranged from 5 to 65,125 persons. Ninety-five FSAs met the inclusion criteria, for which the population size ranged from 2,165 to 65,125 persons (mean = 26,345). A total of 495 laboratory confirmed *S.* Enteritidis infections were identified in the MOHLTC’s iPHIS database during the study period (165 cases in 2007, 168 in 2008, and 162 in 2009). In total, 22 cases (4.4 %) were excluded because of missing FSA data (14 cases in 2007, 4 in 2008, and 4 in 2009). Thus, there were 473 cases (151 in 2007, 164 in 2008, and 158 in 2009) available for analysis. No outbreaks were declared by the MOHLTC during the study period.

### Exploratory spatial analysis

#### Non-smoothed and smoothed standardized incidence rates

**Fig. 3:**
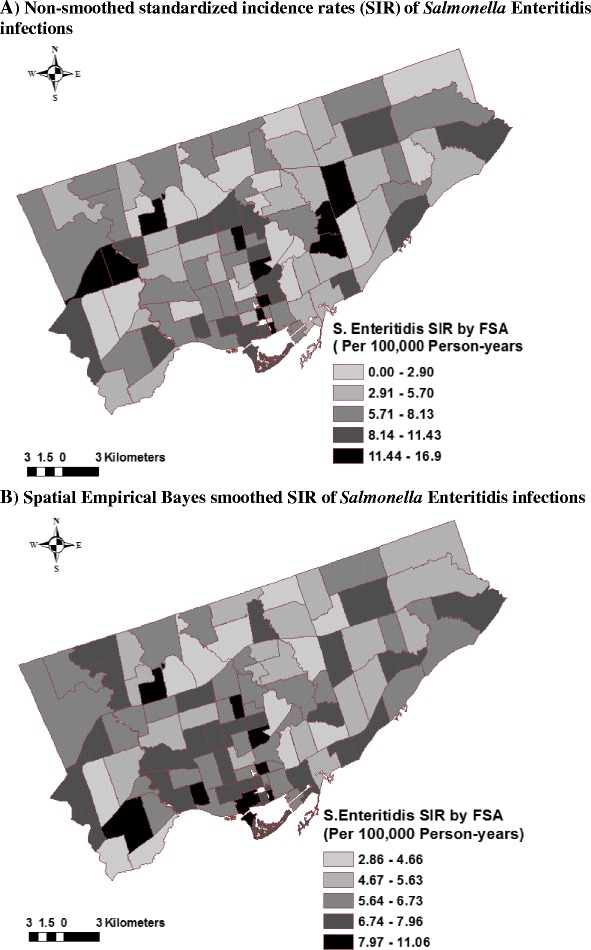
Distribution of non-smoothed (**A**) and smoothed (**B**) *Salmonella* Enteritidis infection rates in Toronto, 2007-2009 (*n* = 473 cases; *n* = 95 forward sortation areas). Direct standardization was used to calculate forward sortation area (**FSA**)-level annual standardized incidence rates (**SIRs**) of *Salmonella* Enteritidis infections. Spatial empirical Bayes smoothing method with 2nd order queen contiguity weights in GeoDa software (Spatial Analysis Lab, University of Illinois Urbana-Champaign, IL, USA) was used to smooth the SIRs. Maps prepared in ArcGIS 10.1 (ESRI Inc., Redlands, CA, USA)

Figure 3 illustrates the non-smoothed and smoothed annual SIRs of *S.* Enteritidis infections per FSA in Toronto. The non-smoothed FSA-level SIRs ranged from 0 to 16.9 infections per 100,000 person-years (mean = 6.6). The smoothed SIRs ranged from 2.9 to 11.1 (mean = 6.3).

### Spatial statistics

#### Global clustering (Getis-Ord General G)

**Fig. 4:**
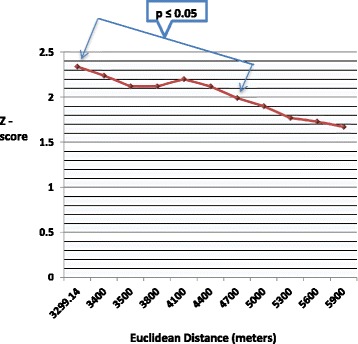
Global clusters of areas with high *Salmonella* Enteritidis infection rates in Toronto at different distances. Results of the Getis-Ord G statistic. Large, positive Z-scores (*e.g.* values ≥ 1.96) indicate global clustering of forward sortation areas with high standardized incidence rates. The zone of indifference conceptualization parameter was used for the analysis. Statistically significant at *p* ≤ 0.05

**Fig. 5:**
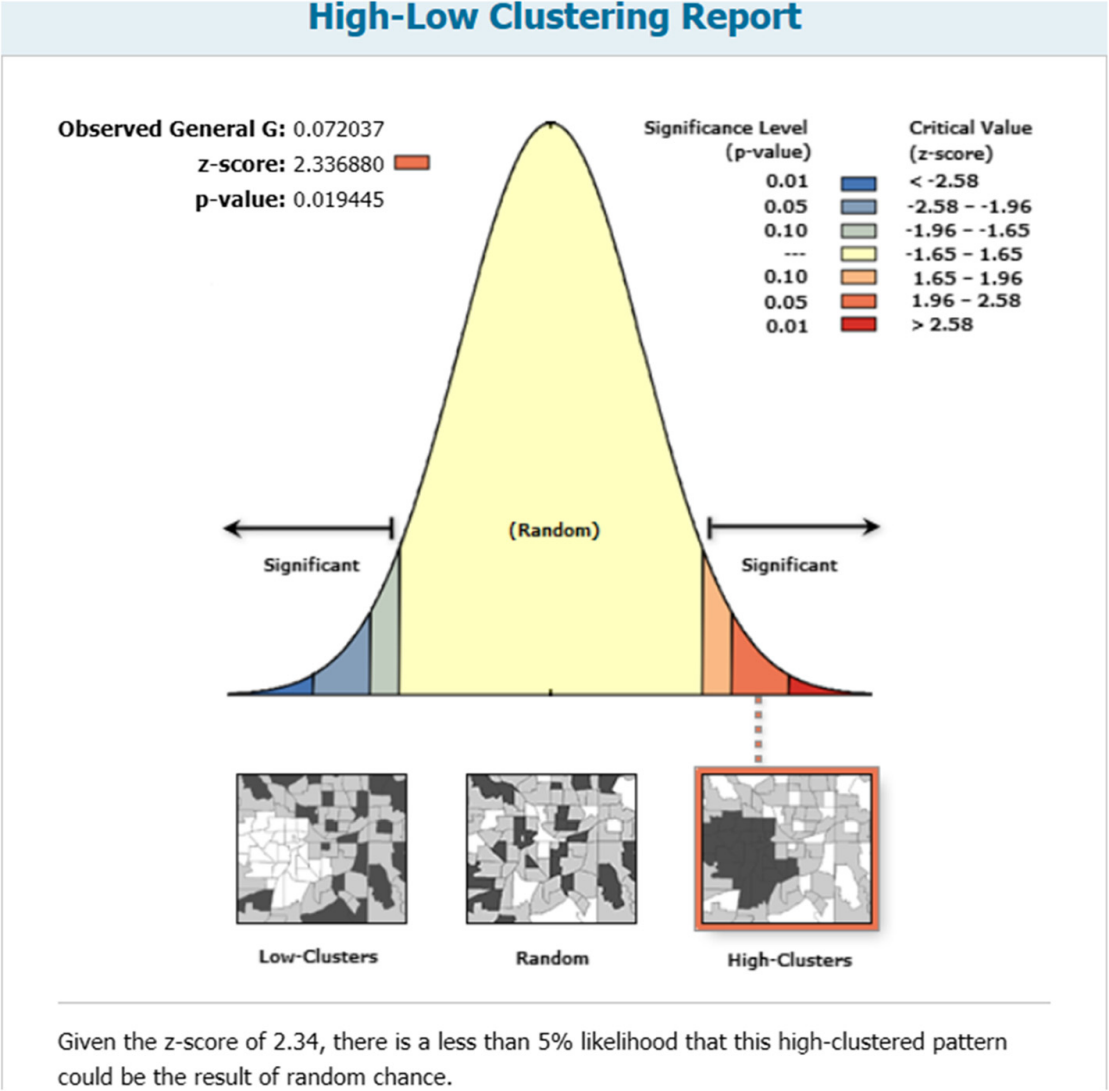
Maximum spatial clustering of areas with high *Salmonella* Enteritidis infection rates in Toronto at 3.3 kilometers. Results of the Getis-Ord G statistic. A large, positive Z-score (values ≥ 1.96) indicates global clustering of forward sortation areas with high standardized incidence rates. The zone of indifference conceptualization parameter was used for the analysis. Statistically significant at *p* ≤ 0.05

The Getis-Ord General G statistic results are shown in Figs. 4 and 5. Statistically significant positive Z-scores (1.99 - 2.34) were observed between 3.3 and 4.7 km. The highest statistically significant positive Z-score was observed at 3.3 km (Z = 2.34, *p* = 0.019), signifying maximum spatial clustering of FSAs with high SIRs at this distance band (Fig. 5). There were no statistically significant negative Z-scores.

### Local clustering

#### Hot spot analysis (Getis-Ord Gi*)

**Fig. 6:**
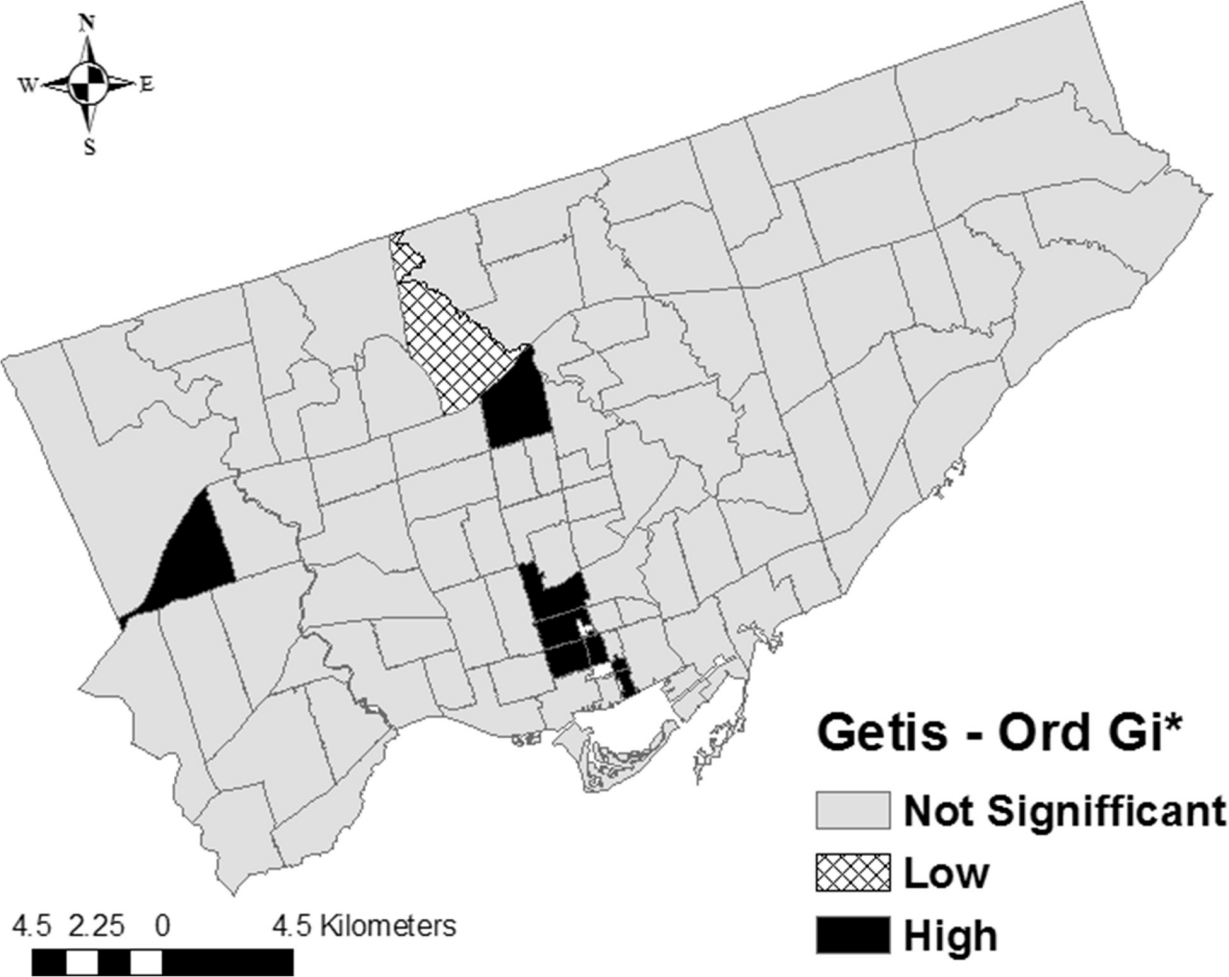
Local clusters of Salmonella Enteritidis infection rates in Toronto identified by the Getis-Ord Gi* statistic. Significant clusters of forward sortation areas (**FSAs**) with high standardized incidence rates (**SIRs**) (Z-score ≥ 1.96; p ≤ 0.05). Significant clusters of FSAs with low SIRs (Z-score ≥ -1.96; *p* ≤ 0.05). A Euclidean distance band of 3.3 km, and the zone of indifference conceptualization parameter, were used for the analysis

Eight FSAs with high SIRs (hot spots) (M5C, M5E, M5G, M5M, M5R, M5S, M5T, M9R) and one FSA with a low SIR (cold spot) (M3H) were detected using the Getis-Ord Gi* method (Table 1, Fig. 6, Additional file 1: Legend 1). The majority of hot spots (6 of 8) were located in south-central (*i.e.* downtown) Toronto.

**Table 1:** Forward sortation areas identified by different local cluster detection methods

Type of cluster	Method	Forward sortation area
High (hot spot)	Getis-Ord Gi*	M5C, M5E, M5G, M5M, M5R, M5S, M5T, M9R
Low (cold spot)	Getis-Ord Gi*	M3H
High-high	Local Moran’s I	M4Y, M5E, M5G, M5M, M9R
Low-low	Local Moran’s I	M3H
High-low	Local Moran’s I	M3M
Low-high	Local Moran’s I	M4G, M5C, M5R

#### Cluster and outlier analysis (Anselin Local Moran’s I)

**Fig. 7:**
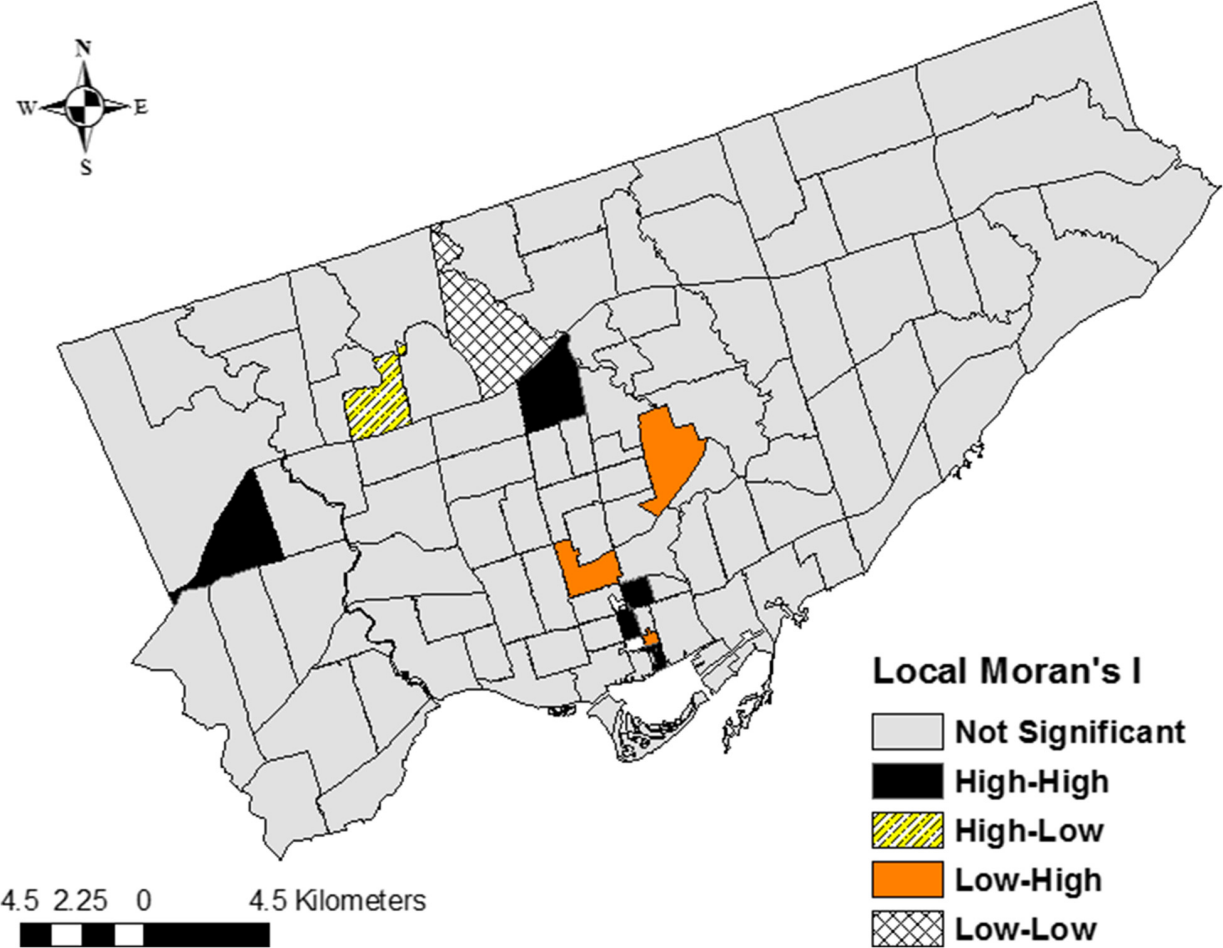
Local clusters of *Salmonella* Enteritidis infection rates in Toronto identified by the Moran’s I statistic. An Euclidean distance band of 3.3 km, and the zone of indifference conceptualization parameter, were used for the analysis. Statistically significant at *p* ≤ 0.05

Five FSAs with high-high SIRs (M4Y, M5E, M5G, M5M, M9R), one FSA with a low-low SIR (M3H), and four outlier FSAs (one high-low (M3M) and three low-high (M4G, M5C, M5R)) were identified using the Local Moran’s I method (Table 1, Fig. 7, Additional file 1: Legend 1). Three FSAs with high-high SIRs were detected in downtown Toronto.

## Discussion

*Salmonella* Enteritidis infection rates clustered globally and locally in the city of Toronto. The small distance band at which high *S.* Enteritidis infection rates clustered globally suggests that infection rates were localized to small distinct areas. This finding was subsequently supported by the local cluster analyses, where distinct FSAs, mainly in downtown Toronto, were identified as areas with significantly high SIRs. The two local cluster detection methods (Getis-Ord Gi* and Local Moran’s I) identified a number of the same clusters, suggesting consistency between these methods, and indicating the robustness of our study results.

We assessed the area-level spatial heterogeneity of *S.* Enteritidis infection rates across the city of Toronto by combining spatial exploratory and spatial statistical methods with GIS. A systematic approach was used, in which analytical steps succeeded each other, starting from more general to more specific stages that increased our study’s specificity. Each step provided additional information to enhance our understanding of the spatial epidemiology of *S.* Enteritidis infection rates in Toronto. However, these steps were sometimes connected and difficult to delineate; consequently, a holistic approach that considers the results of all steps should be followed when interpreting our findings.

The variability of small scale infection rate estimates was accounted for by using the SEB smoothing method. This method reduces the variation of infection rate estimates of areas with unbalanced rates, by shrinking the less stable estimates toward the local mean if local clustering of high-rate areas are detected, and toward the global mean if no local clustering is present [48]. The major advantage of smoothing is that it focuses attention on the overall spatial disease trends, which increases the ability to identify areas with high or low rates. However, as noted with our data, areas can be misclassified by the smoothing method. For example, one high-rate area (M9R) that was evident on the non-smoothed SIR map and subsequently detected by both local cluster detection methods, was hidden by the smoothing process. Fewer FSAs with high rates were identified using the smoothed SIRs compared to the non-smoothed SIRs; nonetheless, in the central and south-central parts of the city, both methods identified many of the same high-rate FSAs. The SEB smoothing method reduced the highest non-smoothed SIR by 5.8 units, indicating that there were FSAs with unstable SIR estimates.

When analyzing small scale area-level data, the spatial estimates can become unbalanced at the study area limits where FSAs do not have neighbours. Moreover, because FSAs’ boundaries are arbitrary delimitations based on the mail distribution zones of postal facilities, they might not always delineate areas based on their spatial characteristics. To account for potential “edge” and “zoning” effects, we used the “zone of indifference” conceptualization parameter, which does not force sharp boundaries on neighbouring FSA’s spatial characteristics nor limit the number of neighbours [52]. This conceptualization parameter considers every FSA to be a neighbour of every other FSA, yet it assigns a maximum weight to areas within a pre-determined distance band, and reduces the intensity of spatial relationships once this distance is passed.

The Getis-Ord G method was valuable for identifying the extent of global clustering. Although Toronto is a large city (area of approximately 630 km^2^), maximum spatial clustering of FSAs with high SIRs was detected at 3.3 km, which suggests that clustering of *S.* Enteritidis infections was localized to relatively small areas within the city. This result might suggest that local clusters were driven by small outbreaks (*e.g.* exposures in homes, local daycares, or restaurants) and not by widespread contamination of food or water supplies. Although outbreak cases are reported to iPHIS and investigated, no local outbreaks were declared by the local public health authorities during the study period.

The Getis-Ord Gi* and Local Moran’s I methods identified several of the same clusters. Specifically, four hot spots (M5E, M5G, M5M, M9R) and one cold spot (M3H) were identified by both methods, highlighting the robustness of our study findings. Moreover, our study results are generally in agreement with our previous study [15], in which we evaluated area-level spatial clustering of *S.* Enteritidis infection rates within three public health units (the City of Toronto, Peel Region, and York Region) in the Greater Toronto Area using a spatial discrete Poisson model within a spatial scan statistic. In that study, a single cluster of significantly higher than expected infection rates located in the south-central part (downtown) of the City of Toronto Health Unit was identified, which included nine neighbouring FSAs (M4Y, M5B, M5C, M5E, M5G, M5S, M5T, M5V, M6J). By comparison, in the current study, the Getis-Ord Gi* method detected five hot spots (M5C, M5E, M5G, M5S, M5T), and the Local Moran’s I method detected three high-high clusters (M4Y, M5E, M5G) and one low-high cluster (M5C) in downtown Toronto. Taken together, these findings show that these spatial methods could be used in real-time for foodborne disease surveillance data analysis or retrospectively for prevention and control program planning.

However, it is important to understand the specifics of each method to avoid making misleading conclusions. The Getis-Ord Gi* method is ideal when there is an assumption that infection rates cluster within the study area, when investigators are only interested in detecting local high- or low-rate clusters, and when there are a limited number of neighbouring areas with dissimilar rates. Because the Getis-Ord Gi* statistic includes the target FSA’s rate when calculating the local sum of rates, it is not as useful in study areas in which there are several small areas with dissimilar rates. For example, if the target FSA has a sufficiently high rate, it can be designated as a hot spot even though it is surrounded by FSAs with low rates. Likewise, some of its neighbouring low-rate FSAs will also be identified as hot spots; or high- or low-rate FSAs will not be identified at all. These issues explain why two of the hot spots (M5C, M5R) identified by the Getis-Ord Gi* method were identified as low-high clusters by the Local Moran’s I method, and why an FSA with a high SIR (M3M) and an FSA with a low SIR (M4G) were undetected by the Getis-Ord Gi* method yet were identified as a high-low and a low-high cluster, respectively, by the Local Moran’s I method. The latter method identifies local areas with dissimilar rates and excludes these from the local high- or low-rate clusters, thus preventing misclassification of FSAs in study areas with relatively high numbers of dissimilar neighbouring areas.

This study was a hypothesis-generating study and did not aim to identify individual-level risk factors that might influence the spatial heterogeneity of *S.* Enteritidis infection rates. However, both demographic and socioeconomic characteristics have been identified as important risk factors for salmonellosis, some of which include eating behaviours (*e.g.* frequency of eating outside the home) [6], international travel patterns of local residents [10, 11], ethnicity (*e.g.* proportion of the population that is non-Caucasian) [12], and the proportion of the population with a high income [12, 13, 15]. Moreover, local clusters of high *S.* Enteritidis infection rates could be explained by differences in environmental contamination of food products in local retail facilities and restaurants [56], variations in microbial quality of food consumed [57], or food safety practices followed by local residents [58]. Future hypothesis-testing studies should be conducted in high-rate FSAs to identify area- and individual-level environmental, behavioural, and socioeconomic risk factors that impact *S.* Enteritidis infection rates. Areas identified as spatial outliers should be investigated using case-control studies (*e.g.* high-rate areas designated as cases and low-rate areas designated as controls) to identify risk factors that contribute to infection rate increases.

As with every population-based ecological study, our research has limitations, which should be considered when interpreting our results. We recognize that analysis at a different scale might offer different results (the “modifiable areal unit problem”) [19, 59]. However, previous studies have demonstrated that examining infection rates at small scales reduces ecological bias, and gives optimal estimates for area-level risk factors for foodborne diseases [19, 60, 61]. The “zoning effect” [62] might also have occurred if neighbourhood boundaries did not follow the area’s spatial characteristics. However, Toronto’s FSAs are of a sufficiently small scale to highlight and delimit neighbourhoods with distinct spatial characteristics, and we also accounted for this issue by using the “zone of indifference” conceptualization parameter. Another limitation of our study is that passive surveillance systems underdiagnose and underreport the true level of infection [2, 63, 64]. Population changes might also have occurred during the study period due to movement of residents into and out of the study area. However, this issue should be minor because populations generally do not change considerably in a relatively short time frame. Lastly, exclusion of cases due to missing information might have affected our results. However, 96 % of available cases were included in our analysis; therefore, our estimates should be reliable.

## Conclusions

To the best of our knowledge, this is the first study worldwide that investigated the spatial epidemiology of *S.* Enteritidis infections in an urban setting. *Salmonella* Enteritidis infection rates clustered globally at a small distance band of 3.3 km, suggesting clustering of high rates in small distinct areas. This finding was supported by the local cluster analyses, where distinct FSAs with high rates, mainly in downtown Toronto, were detected. The robustness of our research findings were demonstrated by linking a number of spatial data explorations and statistical methods with GIS. Our study findings will aid public health professionals to target hypothesis-generating and hypothesis-testing studies in areas with high *S*. Enteritidis infection rates to generate data for public health interventions.

## Additional file

Additional file 1:
**Legend for Table** **1** **and Figs.** **6** **and** **7** **.** Toronto forward sortation area labels. (TIFF 125 kb)

## Abbreviations

FSA: Forward sortation area
GIS: Geographic information system
iPHIS: integrated Public Health Information System
MOHLTC: Ontario Ministry of Health and Long-Term Care
SEB: Spatial empirical Bayes
*S.* Enteritidis: *Salmonella enterica* serovar Enteritidis
SIR: Standardized incidence rate
