# Supplementary figures and images for: Area-level global and local clustering of human Salmonella Enteritidis infection rates in the city of Toronto, Canada, 2007–2009
Source: BMC Infect Dis. 2015 Aug 21;15:359. doi: 10.1186/s12879-015-1106-6 (PMC4545976; doi:10.1186/s12879-015-1106-6)

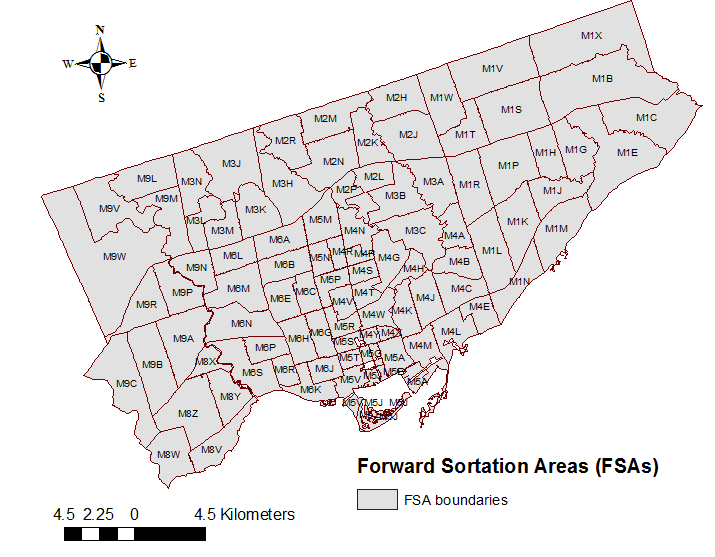

Supplement: Additional file 1: — Legend for Table 1 and Figs. 6 and 7 . Toronto forward sortation area labels. (TIFF 125 kb) [file 12879_2015_1106_MOESM1_ESM.tiff]
